# Phosphoethanolamine cytidylyltransferase ameliorates mitochondrial function and apoptosis in hepatocytes in T2DM in vitro

**DOI:** 10.1016/j.jlr.2023.100337

**Published:** 2023-01-28

**Authors:** Hu Xu, Weizu Li, Lei Huang, Xinyu He, Bei Xu, Xueqing He, Wentong Chen, Yaoxing Wang, Wenjun Xu, Sheng Wang, Qin Kong, Youzhi Xu, Wenjie Lu

**Affiliations:** 1 Basic Medical College, Anhui Medical University, Hefei, China; 2 Center for Scientific Research, Anhui Medical University, Hefei, China

**Keywords:** liver, lipidomics, mitochondria, phospholipids, type 2 diabetes mellitus, phosphatidylethanolamine, phosphatidylserine decarboxylase pathway, cytidine-5′-diphosphate-ethanolamine pathway, high glucose and free fatty acids, ATP synthesis

## Abstract

Liver function indicators are often impaired in patients with type 2 diabetes mellitus (T2DM), who present higher concentrations of aspartate aminotransferase, alanine aminotransferase, and gamma-glutamyl transferase than individuals without diabetes. However, the mechanism of liver injury in patients with T2DM has not been clearly elucidated. In this study, we performed a lipidomics analysis on the liver of T2DM mice, and we found that phosphatidylethanolamine (PE) levels were low in T2DM, along with an increase in diglyceride, which may be due to a decrease in the levels of phosphoethanolamine cytidylyltransferase (Pcyt2), thus likely affecting the de novo synthesis of PE. The phosphatidylserine decarboxylase pathway did not change significantly in the T2DM model, although both pathways are critical sources of PE. Supplementation with CDP-ethanolamine (CDP-etn) to increase the production of PE from the CDP-etn pathway reversed high glucose and FFA (HG&FFA)-induced mitochondrial damage including increased apoptosis, decreased ATP synthesis, decreased mitochondrial membrane potential, and increased reactive oxygen species, whereas supplementation with lysophosphatidylethanolamine, which can increase PE production in the phosphatidylserine decarboxylase pathway, did not. Additionally, we found that overexpression of PCYT2 significantly ameliorated ATP synthesis and abnormal mitochondrial morphology induced by HG&FFA. Finally, the BAX/Bcl-2/caspase3 apoptosis pathway was activated in hepatocytes of the T2DM model, which could also be reversed by CDP-etn supplements and PCYT2 overexpression. In summary, in the liver of T2DM mice, Pcyt2 reduction may lead to a decrease in the levels of PE, whereas CDP-etn supplementation and PCYT2 overexpression ameliorate partial mitochondrial function and apoptosis in HG&FFA-stimulated L02 cells.

Type 2 diabetes mellitus (T2DM) is the most common type of diabetes, accounting for approximately 90% of all diabetes cases (1). Over time, it can cause damage to the heart, blood vessels, eyes, kidneys, and nerves (2). T2DM is a serious problem worldwide, with 700 million people expected to be affected by the disease by 2045 (3). Epidemiological statistics show that T2DM is closely related to metabolic syndromes, such as hypertension, hyperlipidemia, and obesity (4). Nonalcoholic fatty liver disease and nonalcoholic steatohepatitis are common in patients with T2DM but the relationship between liver metabolic disorders and T2DM is complicated (5, 6, 7). Impaired liver function indicators are often observed in patients with T2DM, with higher aspartate aminotransferase (AST), alanine aminotransferase (ALT), and gamma-glutamyl transferase concentrations in subjects with T2DM than in those without T2DM (8). In addition, pathological tissue sections showed significant steatosis in the livers of patients with T2DM (9). The level of liver inflammation is significantly increased in patients with T2DM, and insulin resistance models in HepG2 cell lines show oxidative stress, increased levels of reactive oxygen species (ROS), and increased apoptosis (10). Mitochondria are the most important energy metabolism sites of cells, and under hyperglycemic conditions, the oxidative phosphorylation ability of mitochondria decreases. An increase in electron transport chain and ROS production is also believed to aggravate pathological processes. Therefore, diabetic patients often have mitochondrial dysfunction, but the specific molecular mechanism of mitochondrial dysfunction caused by T2DM has yet not been fully elucidated (11).

Phospholipids are an integral part of biological membranes and are important signaling molecules involved in many physiological processes, thus, they are vital for maintaining body homeostasis (12). The second most abundant phospholipid in the human body is phosphatidylethanolamine (PE), which is involved in various cell processes including apoptosis, autophagy, and membrane fusion (13). There are four synthetic pathways of PE in eukaryotes, of which the two most important are the de novo synthesis of PE by the CDP-ethanolamine (CDP-etn) pathway and phosphatidylserine decarboxylase (PISD) pathway (14). Some studies have shown that PE levels in the livers of mice with T2DM diabetes are significantly reduced but the specific mechanism of such phospholipid changes remains unclear (15, 16).

Phosphoethanolamine cytidylyltransferase (PCYT2) is generally considered to be a rate-limiting enzyme in the CDP-etn pathway (17). PCYT2 plays an important role in liver PE synthesis. In fact, most PE is synthesized by the CDP-etn pathway on the endoplasmic reticulum (ER). In CDP-etn pathway, cells first convert the ingest ethanolamine into phosphoethanolamine through ethanolamine kinase, which is converted into CDP-etn under the catalysis of PCYT2, and finally, CDP-etn and diglyceride (DG) form PE under the catalysis of CDP-etn: 1,2-diacylglycerol ethanolamine phosphotransferase (18). It was confirmed that low expression of Pcyt2 in the liver can lead to severe accumulation of diglycerol ester and hepatic steatosis (19). In addition, Pcyt2 inhibition results in mitochondrial respiratory dysfunction caused by the accumulation of its substrate, phosphoethanolamine, in MCH58 fibroblasts (20). These findings indicate that PCYT2 is indispensable to the maintenance of lipid metabolism and mitochondrial homeostasis.

Based on this evidence, we investigated whether PCYT2 is involved in liver injury induced by T2DM and its possible molecular mechanism. This study opens new avenues for the treatment of liver damage in patients with T2DM.

## MATERIALS and METHODS

### Reagents

AST and ALT testing kits were purchased from Nanjing Jiancheng Biotechnology Institute (Nanjing, China). Lyso PE (18:1) (846725P), CDP-etn (90,756), oleic acid (OA; O1008), and palmitic acid (PA; P0500) were purchased from Sigma (Darmstadt, Germany). An ATP assay kit (S0026), BCA protein assay kit (P0010S), mitochondrial membrane potential assay kit (C2006), and ROS assay kit (S0033S) were obtained from Beyotime (Shanghai, China). RPMI 1640 medium (SH30027.01) and FBS (SH30406.02) were acquired from Gibco (Waltham, MA). PCYT2 (ab135290) antibody was supplied by Abcam (Cambridge); BAX (#5023), Bcl-2 (#3498), cleaved-caspase3 (#9661), and β-actin (#3700) were supplied by Cell Signaling Technology (Danvers, MA); and PISD (sc-390070) was supplied by Santa Cruz Biotechnology (Santa Cruz, CA). Elabscience (Shanghai, China) supplied goat anti-rabbit (E-AB-1034) and goat anti-mouse (E-AB-1035) secondary antibodies. TRIzol reagent (15,596,018) and RevertAid RT kit (K1691) were obtained from Thermo Fisher Scientific (Waltham, MA). The primers for qRT-PCR were synthesized by Sangon Biotech (Shanghai, China). A SYBR green fluorescent probe was obtained from Bio-Rad (Richmond, CA).

### Cell culture and experiments

An established human fetal hepatocyte line (L02) was received from the ATCC. RPMI 1640 medium with 10% FBS and 1% penicillin/streptomycin were used to culture the cells. The incubator conditions used were 5% CO_2_ and 37.0°C. To assess the effect of high glucose (HG) with free fatty acids (FFA) on cell lipid metabolism, cells were exposed to 30 mM D-glucose with 1 mM FFA (OA:PA = 2:1) as described previously (21). In order to detect the effects of related substances on cells respectively, lysophosphatidylethanolamine (LPE) (100 μM) and CDP-etn (100 μM) were added to the medium within the HG&FFA mix. CDP-etn refers to CDP-etn sodium salt hydrate; it was dissolved in culture medium and supplied to cells. LPE was dissolved in ethanol and then added to the culture medium and provided to the cells.

### Gene transfection

The lentivirus for overexpression of *Homo sapiens* PISD and PCYT2 was purchased from GENECHEM (Shanghai, China). The vector backbone used was GV492, the sequence was Ubi-MCS-3FLAG-CBh-gcGFP-IRES-puromycin, and the cloning site was BamHI/AgeI. The control cells were infected with an equal quantity of negative control virus named CON335 (Ubi-MCS-3FLAG-CBh-gcGFP-IRES-puromycin). To generate stable cell lines, lentiviruses were incubated overnight with cells, and then the cells were sorted based on the presence of GFP. Next, puromycin was used to select stably transfected cells. Stably transfected L02 cells were used for the following experiments.

### Animal model

Establishment of the T2DM diabetes model in mice was achieved as previously described (22). C57BL/6J mice aged 8 weeks were used in this study. The cages were maintained in a specific pathogen-free, temperature-controlled environment with alternating light and dark 12-h period. The animals had free access to water and food for one week as they acclimated. The animals were then randomly assigned to a normal diet group (CON, Cat. 1010009) or a high-fat diet group (HFD, 60% energy from fat, Cat. XTHF60-1) for the following 8 weeks. Mice in the HFD group were made to fast overnight and then were intraperitoneally injected with streptozotocin (STZ) citric acid buffer (pH 4.0) at 40 mg/kg/d each day for seven consecutive days. Seven days later, the blood glucose level of each animal was detected. Animals with a fasting blood glucose level greater than 11.1 mM were considered as T2DM mice. At the end of the experiment, the mice were anesthetized with sodium pentobarbital at a dose of 100 mg/kg; the mice were fasted overnight prior to sacrifice. The mice were then dissected and the blood and liver were collected. The animal experiments were approved by the Laboratory Animal Ethics Committee of Anhui Medical University (Hefei, China) and carried out in accordance with the Guidelines for the Care and Use of Laboratory Animals.

### RNA isolation and real-time PCR

Total RNA was extracted from mouse liver tissue and cells using TRIzol reagent. RNA was quantified using a nucleic acid concentration detector (Molecular Devices, Sunnyvale, CA) at 260 nm (OD260). The mRNA in total RNA was reversed into cDNA using a RT kit and a gradient PCR instrument (Bio-Rad, Hercules, CA) as per the manufacturer’s protocol. A real-time fluorescence quantitative PCR instrument (Bio-Rad) was used to detect mRNA expression levels and the β-actin gene was used as the reference gene. The results of the relative gene expression levels were normalized, supplemental Table S1 shows the primer sequences used for qRT-PCR analyses.

### Western blot analysis

Total protein was extracted from cells and liver tissue using RIPA buffer supplemented with phosphatase and protease inhibitors. Equal amounts of protein from each sample were electrophoresised in 8–12% SDS-PAGE and then transferred to PVDF membranes (Merck, Darmstadt Germany). The membrane was blocked with 5% skimmed milk in tris-buffered saline solution and Tween-20 (TBST) and then incubated overnight in the following primary antibodies: anti-PCYT2 (1:250), anti-PISD (1:500), anti-BAX (1:500), anti-Bcl-2 (1:500), anti-cleaved-caspase3 (1:500), and anti-β-actin (1:5000). Then, the blots were incubated with the corresponding secondary antibodies conjugated with horseradish peroxidase. Western blotting was observed using a chemiluminescence solution (Affinity, Changzhou, China) and a protein imager (Bio-Rad).

### Oral glucose tolerance and insulin tolerance tests

After two groups of mice were fasted overnight, an oral glucose tolerance test (OGTT) and an insulin tolerance test (ITT) were performed. In the OGTT experiment, the mice were intragastric administration with glucose solution (2 g/Kg). The ITT experiment was performed one week after the completion of the OGTT experiment. An insulin solution (prepared with 0.5 U/kg physiological saline) was intraperitoneally injected, and glucose test strips were used to measure the blood glucose concentration at 0, 15, 30, 60, and 120 min following injection.

### Preparation of lipidomics samples

Frozen liver tissue was removed from a refrigerator maintained at −80°C. Surgical scissors were used to cut 50 mg of frozen tissue and placed in the eppendorf (EP) tube. Physiological saline (0.9% NaCl) was added to the tissue at a ratio of 10:1. After grinding with a tissue grinder for 1 min, 600 μl of the homogenate was placed to another EP tube. Then, 800 μl of methyl *tert*-butyl was added, vortexed, and mixed for 1 min. The mixture was shaken at 1500 rpm for 30 min at 25°C. Then, the mixture was centrifuged at 12,000 *g* at 4°C for 10 min and then left to stand for 5 min to separate the solution. Five hundred microliters of the upper layer solution was then pipetted into another EP tube and nitrogen was blown in until the solution was completely volatilized. For reconstitution, 200 μl of isopropanol/methanol 1:1 (v/v) was added, vortexed, and mixed for 5 min. This reconstituted solution was then extracted using syringes, passed through a 0.22 μm filter, and then transferred into lighttight sample vials.

### Lipid profiling

Liquid chromatographic conditions were as follows: Acclaim C30 column (3.0 μm, 2.1 mm × 150 mm); column temperature: 45°C; flow rate: 0.26 ml/min; injection volume: 5 μl. Mobile phase A was ethylene glycol/water (60:40, v/v, containing 0.1% formic acid and 0.1% ammonium formate), and B was isopropyl alcohol/ethylene glycol (90:10, v/v, containing 0.1% formic acid and 0.1% ammonium formic acid). Liquid chromatography gradient elution program was as follows: 30% B over 2 min, 43% B from 2 to 2.1 min, 55% B from 2.1 to 12 min, 65% B from 12 to 18 min, 100% B from 18 to 25 min and then 30% B from 25 to 30 min. Mass spectrometry conditions were as follows: ion source: electron spray ionization (ESI) source; spray voltage: 3800/3200 V (+/−); ionization modes: ESI^+^, ESI^-^; sheath gas: 40 arb; auxiliary gas: 10 arb; capillary temperature: 320°C; scanning range: 100–1500 m/z; resolution: 17,500 full width at half-maximum. Nitrogen was used as the carrier gas.

### PE content determination

L02 cells were cultured in Petri dishes with 10 cm diameter. After exposure to different treatments, mitochondria of L02 cells were isolated using a cell mitochondria isolation kit (Beyotime, Shanghai, China) according to the manufacturer’s instructions. The mitochondrial and extramitochondrial contents were homogenized in 5% Triton X-100 and samples were heated at 80°C to solubilize all lipids. The mitochondrial and extramitochondrial PE content was determined using a PE assay kit (MAK361, Merck KGaA, Germany). Thereafter, 10 μl of each sample (or standard) and Working Reagent Mix were added to each well of a 96-well plate. The fluorescence intensity was measured using an automatic microplate reader (Varioskan Flash, Thermo Fisher Scientific) at excitation and emission wavelengths of 535 and 587 nm, respectively.

### H&E staining

The livers were fixed with 4% paraformaldehyde and then embedded in paraffin wax. Kept the thickness of the slice was 4 μm using a histotome. A H&E staining kit (Servicebio, Wuhan, China) was used for the experiments according to the protocol.

### ROS detection

L02 cells were inoculated into 6-well plates at 1 × 10^6^ per well and treated with HG&FFA, CDP-etn, or LPE. The cell culture medium was removed from the plates, and the cells were then incubated in 1 ml of medium containing 2',7'-dichlorodihydrofluorescein diacetate, 10 μM for 20 min in a cell culture box. After incubation, the cells were washed three times with serum-free cell culture medium to fully remove the probe that did not enter the cells. The cells were then observed using a fluorescence microscope (Carl Zeiss AG, Oberkochen, Germany). The fluorescence intensity was analyzed using ImageJ software (National Institutes of Health, Bethesda, https://imagej.net/ij/index.html).

### Mitochondrial membrane potential detection

L02 cells were plated with 1 × 10^5^ cells per well in 24-well plates. After HG&FFA, CDP-etn, or LPE treatment, the cells were incubated with 200 μl serum-free medium with a dissolved JC-1 probe (5 μg/ml) at 37°C for 20 min then washed twice with a staining buffer (1×). After washing, 500 μl of culture medium was added to each well. The cells were observed using a fluorescence microscope and the fluorescence intensity was analyzed using ImageJ software.

### ATP detection

The ATP content in the cells was detected using an ATP kit (Beyotime, Shanghai, China). A sample tube containing 20 μl of sample or standard substance was placed in a luminometer (SuPerMax 3100) and mixed quickly with a micropipette. After a minimum of 2 s interval, the relative lights unit value was measured.

### Apoptosis detection

L02 cells were plated in six-well plates at 2 × 10^5^ cells per well. After treatment with HG&FFA, CDP-etn, or LPE, the cells were collected in EP tubes, stained with 2.5 μl Annexin V-FITC and 2.5 μl propidium iodide, incubated at 25°C for 10–20 min, mixed well, and then detected by flow cytometry.

### Electron microscope

The cells were cultured in Petri dishes each with a diameter of 6 cm. After treatment, the cells were collected and counted. Samples containing 1 × 10^6^ cells were fixed by slowly pouring them into 2.5% glutaraldehyde fixative solution along the tube wall and storing in a refrigerator at 4°C. Ethanol gradient dehydration (30, 50, 70, 80, 90, and 100%), critical point drying, and coating were performed followed by electron microscope (EM) observation.

### Statistical analysis

Two-tailed unpaired and paired *t*-tests were used to determine whether the outcomes were significantly different between the two groups. For statistical analysis of more than two groups, ANOVA was used. Results are mean ± SD. *P* < 0.05 was considered a statistically significant.

## RESULTS

### Mice with T2DM have impaired liver function and altered lipid composition

**Fig. 1:**
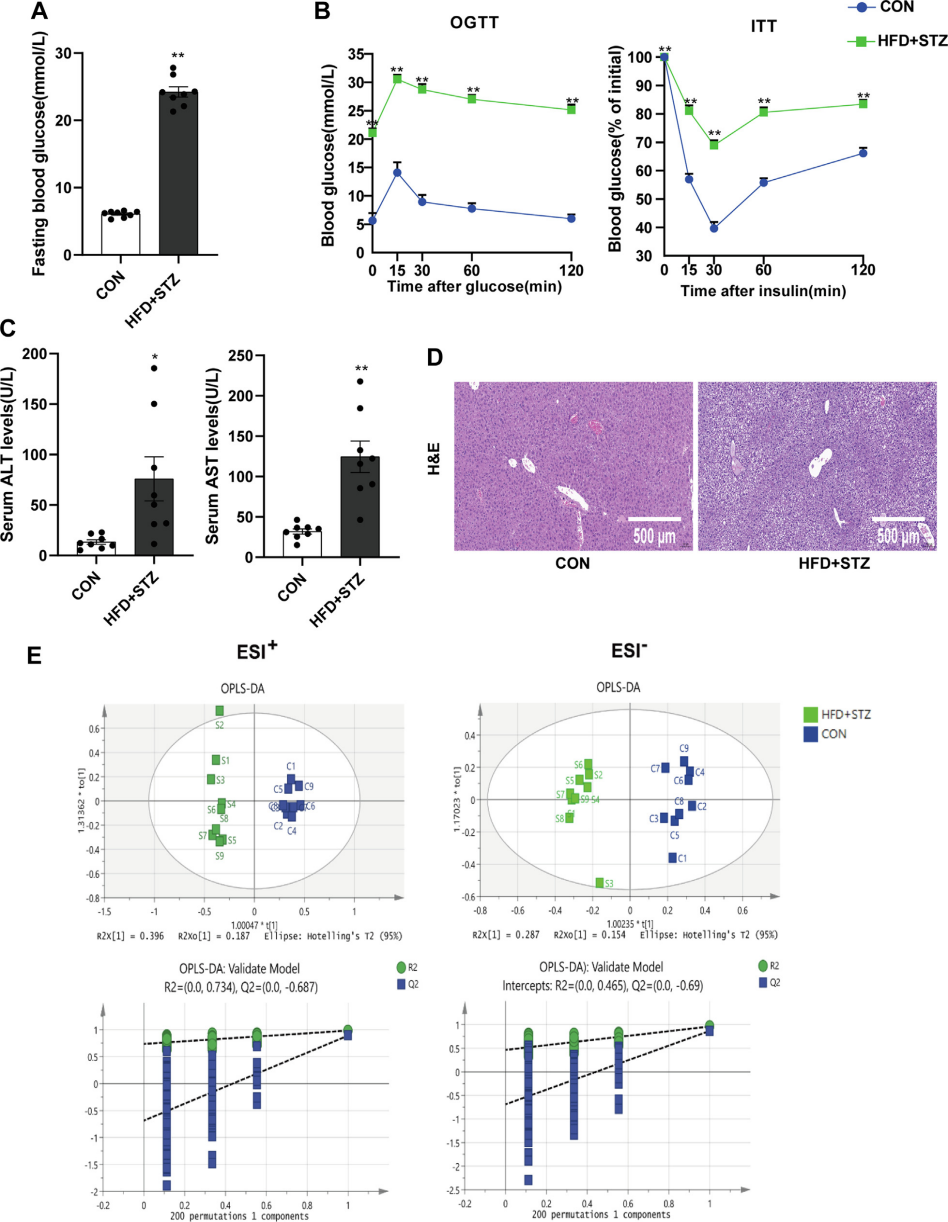
Impaired liver function and liver lipidomics in diabetic mice. A: Fasting blood glucose level of mice (n = 8). B: OGTT and ITT (n = 8). C: Serum ALT and AST level of mice (n = 8). D: Liver histology determined by H&E. E: OPLS-DA score plots of lipidomics, comparing CON and HFD+STZ groups, as well as the validation of OPLS-DA models by permutation analysis (200 times) (n = 9). All experiment were repeated three times. ∗*P* < 0.05, ∗∗*P* < 0.01. ALT, alanine aminotransferase; AST, aspartate aminotransferase; HFD, high-fat diet; ITT, insulin tolerance test; OGTT, oral glucose tolerance test; OPLS-DA, orthogonal projections to latent structures discriminant analysis; STZ, streptozotocin.

The fasting blood glucose level of the T2DM (HFD + STZ) mice was considerably higher than that of the control (CON) group (Fig. 1A). The OGTT showed that the fasting blood glucose level of the T2DM mice was almost four times higher than that of the CON group; meanwhile, the area under the curve for the model group was 53.89 mmol/L·h, while that for the CON group was 16.39 mmol/L·h. The ITT indicated that the effect of insulin in lowering blood glucose on the HFD + STZ group was significantly lower than on the CON group (Fig. 1B). Compared with the CON group, the levels of serum ALT and AST were significantly higher in the HFD + STZ group, and H&E staining showed HFD + STZ mice had significant hepatic steatosis (Fig. 1C, D), indicating that liver function was impaired. The lipids in the liver tissues of the CON and HFD + STZ mice were analyzed and identified by lipid chromatography and mass spectrometry. After the data were normalized by the total peak area, an unsupervised principal component analysis was used to analyze the data, which showed that the model sample was well separated from the control group. On this basis, two supervised separation methods, partial least squares discriminant analysis (PLS-DA), and orthogonal projections to latent structures discriminant analysis (OPLS-DA) were used to improve the separation effect. The OPLS-DA model was verified by permutation analysis (200 times). The results indicated satisfactory predictive ability (Fig. 1E).

### CDP-etn pathway is impaired in T2DM mice liver

**Fig. 2:**
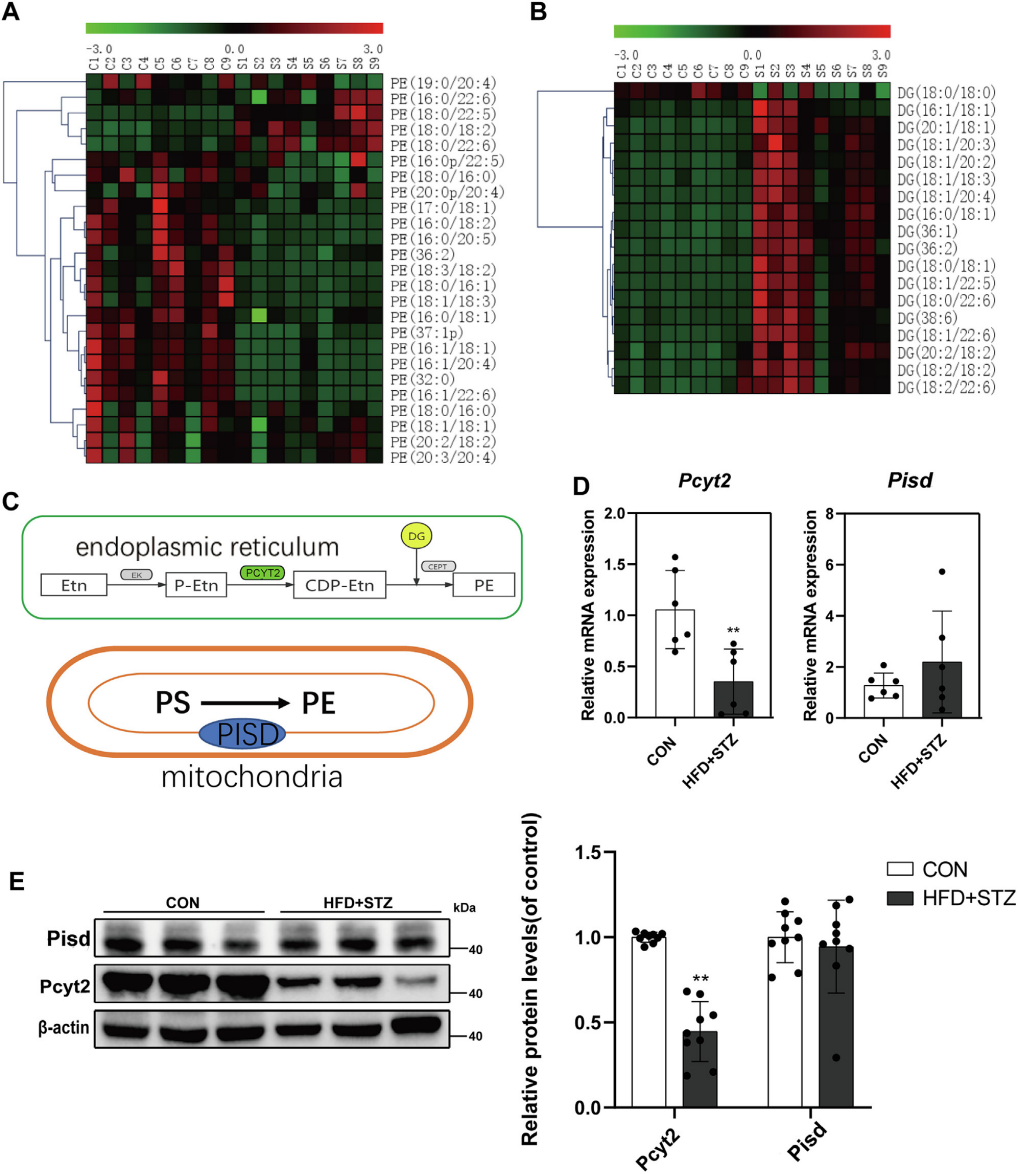
Metabolites and enzymes related to PE de novo synthesis pathway changed significantly in the liver of T2DM mice. A and B, Heat maps of PE (A) and DG (B) in the liver tissues. C: Two major pathways of PE synthesis in mammalian cells, the PISD pathway and de novo pathway. D: qRT-PCR was used to detect mRNA levels of *Pcyt2* and *Pisd* in mice liver (n = 6). E: The protein levels of Pcyt2 and Pisd in mice liver were detected by Western blotting (n = 3). All experiments were repeated three times. ∗*P* < 0.05, ∗∗*P* < 0.01. DG, diglyceride; Pcyt2, phosphoethanolamine cytidylytransferase; PE, phosphatidylethanolamine; Pisd, phosphatidylserine decarboxylase; qRT-PCR, quantitative RT-PCR; T2DM, type 2 diabetes mellitus.

In the lipidomics data, variable important in projection values greater than 1 and values of the *t* test lower than 0.05 were defined as metabolic differences. Among the many different metabolites, the PE content in the liver of the HFD + STZ group was observed to be lower than that of the CON group (Fig. 2A). Therefore, we tested the key enzymes in two main PE synthesis pathways: PISD in the mitochondria and PCYT2 in the ER, respectively (Fig. 2C). The mRNA and protein levels of Pcyt2 in the livers of T2DM mice were significantly decreased, while the mRNA and protein levels of Pisd did not change significantly (Fig. 2D, E). We found that the DG level in the liver of HFD + STZ mice was significantly high (Fig. 2B). DG is the substrate of PCYT2, which catalyzes phosphoethanolamine to produce CDP-etn. This indicates that the change in DG may be due to the decreased levels of Pcyt2.

### HG&FFA treatment impaired the CDP-etn pathway in hepatocytes in vitro

**Fig. 3:**
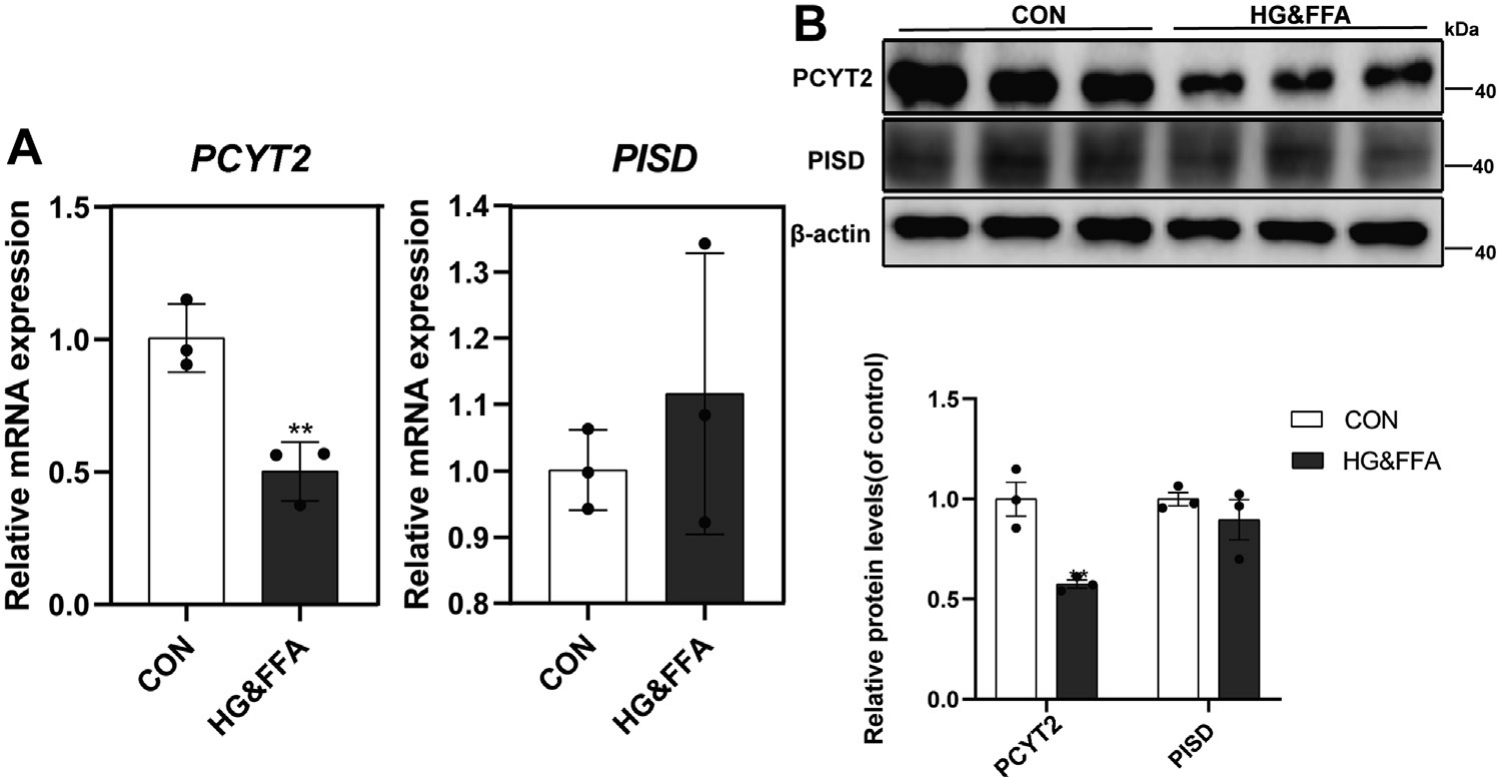
Related metabolic enzymes in PE de novo synthesis pathway in L02 cells were significantly changed by high glucose and free fatty acids stimulation. A: qRT-PCR was used to detect mRNA levels of *PCYT2* and *PISD* in L02 cells (n = 3). B: Western blotting was used to detect protein levels of PCYT2 and PISD in L02 cells (n = 3). All experiments were repeated three times. ∗*P* < 0.05, ∗∗*P* < 0.01. PCYT2, phosphoethanolamine cytidylytransferase; PE, phosphatidylethanolamine; PISD, phosphatidylserine decarboxylase; qRT-PCR, quantitative RT-PCR.

To further understand the mechanism of liver lipid metabolism disorders in T2DM, we tested the mRNA and protein levels of PCYT2 and PISD in L02 cells under HG&FFA stimulation. The PCYT2 expression was significantly reduced, while the mRNA and protein levels of PISD did not change significantly (Fig. 3A, B). Based on these results, we next tested glucose intake in L02 cells treated with glucosamine (18 mM), which is an insulin resistance inducer (supplemental Fig. S2A). These cells showed a significant reduction in glucose intake when compared with that of control cells. In addition, the protein and mRNA levels of PCYT2 in L02 cells treated with glucosamine decreased significantly, while those of PISD did not change (supplemental Fig. S2B, C). These results were consistent with our observations in the livers of T2DM mice where Pcyt2 levels were lower than that in control mice while the levels of PISD did not change.

### Addition of CDP-etn and overexpression of PCYT2 reversed the changes in lipid metabolites in L02 cells stimulated by HG&FFA

**Fig. 4:**
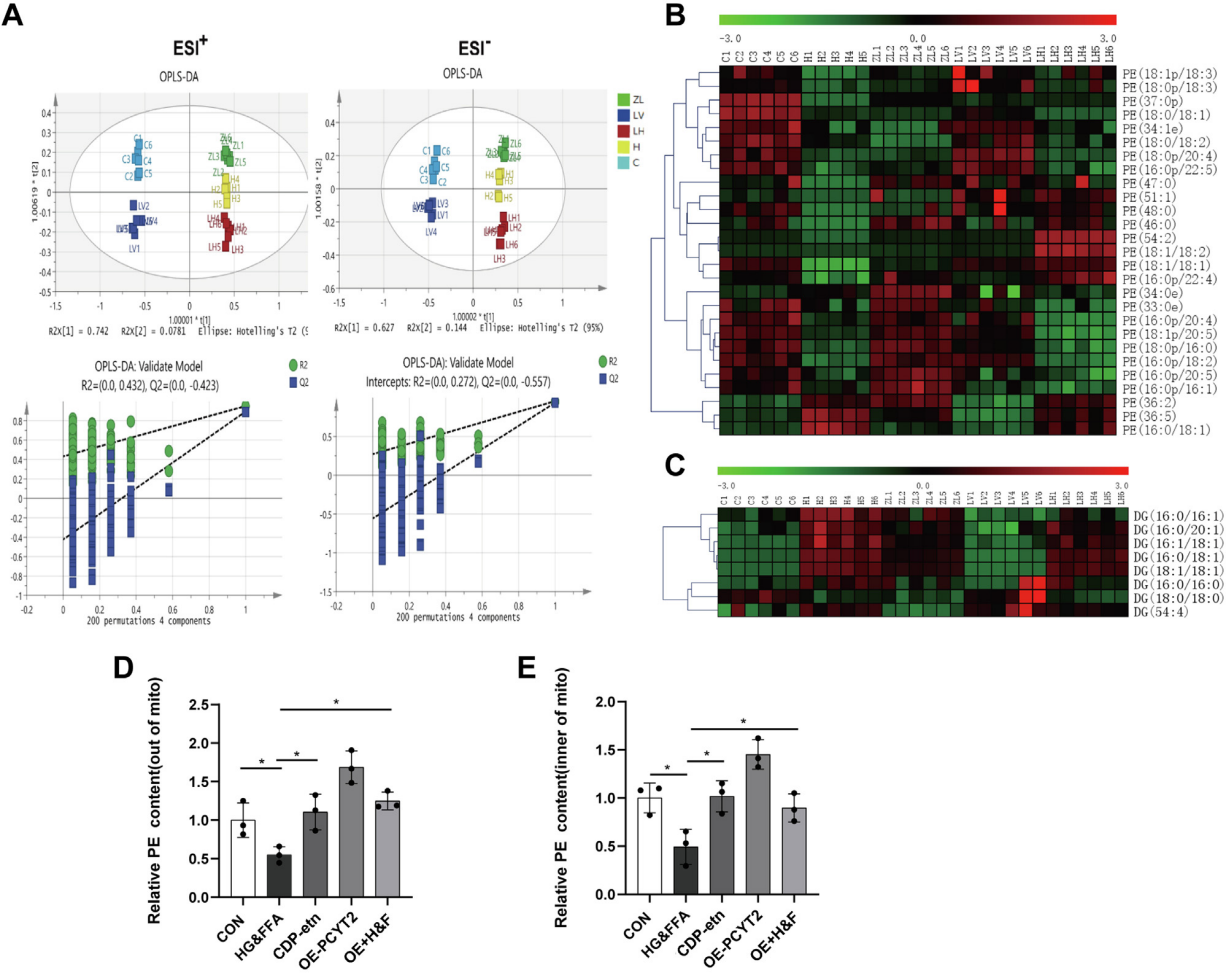
CDP-etn supplementation or overexpression of PCYT2 reversed the changes of L02 metabolites caused by HG&FFA. A: OPLS-DA score plots of lipidomics as well as the validation of OPLS-DA models by permutation analysis (200 times) in L02 cells (n = 5–6). B and C, Heat maps of PE (B) and DG (C) in L02 cells. D and E, The mitochondrial and extramitochondrial PE content in different groups of cells (CON, control group; HG&FFA, high glucose and free fatty acids; CDP-etn, CDP-etn supplementation in L02 cells stimulated with HG&FFA; OE-PCYT2, overexpression of PCYT2 group; OE-PCYT2+H&F, overexpression of PCYT2 in L02 cells stimulated with HG&FFA, n = 3). ∗*P* < 0.05, ∗∗*P* < 0.01. DG, diglyceride; CDP-etn, CDP-ethanolamine; HG&FFA, high glucose and free fatty acids; OE PCYT2, PCYT2 overexpression; OPLS-DA, orthogonal projections to latent structures discriminant analysis; PE, phosphatidylethanolamine; PCYT2, phosphoethanolamine cytidylytransferase.

To further confirm the effect of PCYT2 on liver metabolites in T2DM, we tested the lipids of control L02 cells (C), HG&FFA-stimulated L02 cells (H), HG&FFA-stimulated L02 cells with 100 μM CDP-etn (ZL), L02 cells overexpressing PCYT2 (LV), and L02 cells overexpressing PCYT2 under HG&FFA stimulation (LH). CDP-etn is a direct product of PCYT2 catalysis. Addition of CDP-etn can specifically supplement the PE produced by the CDP-etn pathway (23). After the data were normalized by the total peak area, unsupervised principal component analysis was used to separate the model samples from the control group. On this basis, two supervised separation methods, PLS-DA and OPLS-DA, were used to improve the separation effect. The OPLS-DA model was also verified by a permutation analysis (200 times) (Fig. 4A). The results indicated satisfactory predictive ability. Variables with important in projection values greater than 1 and *P* < 0.05, were defined as metabolic differences. The PE and DG were converted into heatmaps. The results showed that under HG&FFA stimulation, the PE content in L02 cells significantly decreased and the DG content increased significantly, but CDP-etn treatment significantly reversed the PE and DG levels caused by HG&FFA. Compared with control L02 cells, the PE and DG levels of PCYT2 overexpression (OE-PCYT2) cells did not change significantly, but OE-PCYT2 cells showed a reversal of the increase in DG levels and the decrease in PE levels caused by HG&FFA (Fig. 4B, C).

Furthermore, we have demonstrated that PCYT2 levels in the hepatocytes of T2DM model were lower than those in the control group, in vivo and in vitro, and low PCYT2 levels resulted in a decrease in PE levels. To explore whether PE content in the mitochondria was also reduced under diabetic conditions, we extracted the mitochondria and determined the mitochondrial and extramitochondrial PE contents. As a result, the PE content of L02 cells under HG&FFA stimulation was reduced to half of that of the control group both inside and outside mitochondria, however, after supplementation with CDP-etn, the PE content returned to normal levels. Overexpression of PCYT2 in L02 cells increased the PE content significantly, with the higher increase being observed outside the mitochondria. Under HG&FFA stimulation, PCYT2 overexpression ameliorated the PE content and the effect was more significant outside the mitochondria (Fig. 4D, E). This phenomenon may be attributed to the flow of phosphatidylserine (PS) and PE between the mitochondria and ER: PS is transformed into PE in the mitochondria, while PE is transformed into PS in the ER, although PCYT2 is expressed outside the mitochondria. These results indicate that under cellular stimulation by HG&FFA, PCYT2 plays an important role in regulating the mitochondrial and extramitochondrial PE content.

### HG&FFA affect the mitochondrial function of L02 cells through the CDP-etn pathway

**Fig. 5:**
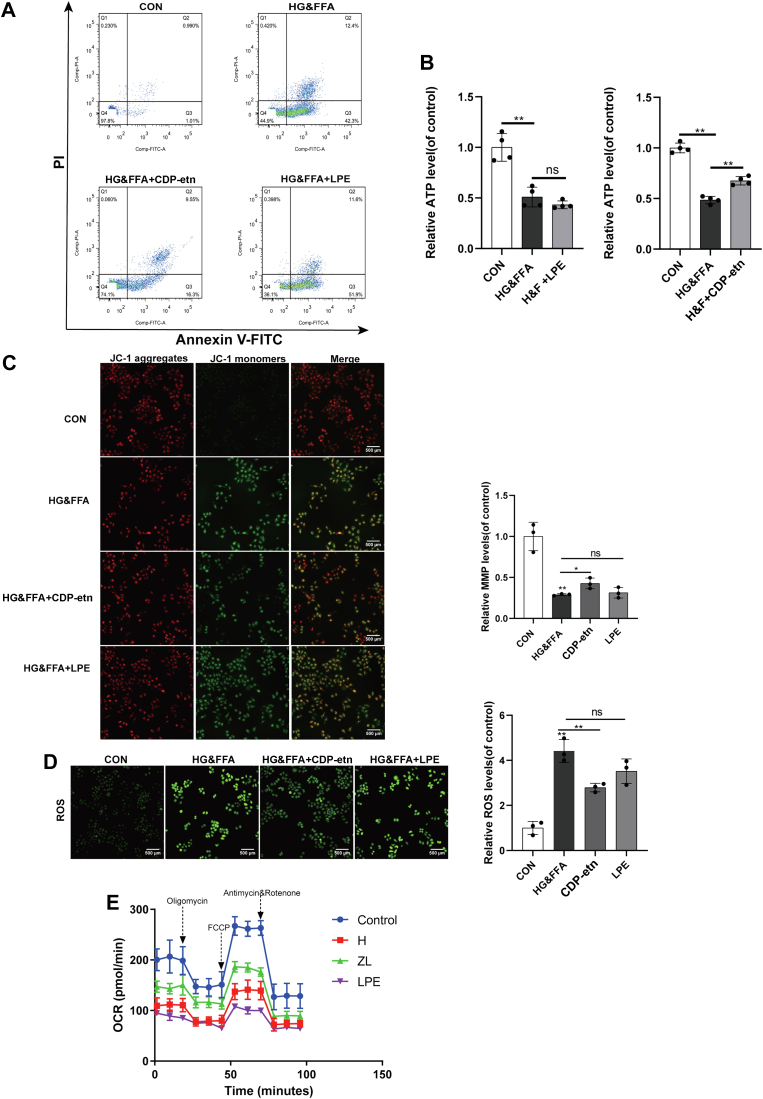
HG&FFA affect the mitochondrial function of L02 cells through the CDP-ethanolamine pathway. A and B, The apoptosis rate (A) and ATP content (B) of L02 cells stimulated by HG&FFA with or without LPE and CDP-etn (n = 4). C: The changes of mitochondrial membrane potential in each group was detected by JC-1 probe in L02 cells (n = 3). D: DCFH-DA probe was used to detect ROS levels in L02 cells (n = 3). E: The mitochondrial oxygen consumption rate of L02 cells was detected using Seahorse (H, L02 cells stimulated by HG&FFA; ZL, CDP-etn supplementation in L02 cells stimulated with HG&FFA; LPE, LPE supplementation in L02 cells stimulated with HG&FFA, n = 3). All experiments were repeated three times. ∗*P* < 0.05, ∗∗*P* < 0.01. DCFH-DA, 2',7'-dichlorodihydrofluorescein diacetate; HG&FFA, high glucose and free fatty acids; LPE, lysophosphatidylethanolamine; ROS, reactive oxygen species.

Mitochondrial PE is mainly provided by PISD, so supplementation with LPE can alleviate the decrease in PE caused by the decrease in PISD expression (24). To investigate the role of PCYT2 in maintaining mitochondrial function, the effects of LPE or CDP-etn on the mitochondrial function and apoptosis level of L02 cells stimulated by HG&FFA were compared. The results of flow cytometry showed that after adding 100 μM CDP-etn, the apoptosis rate was changed from 38% in the model group (HG&FFA) to 18%, which effectively reduced the rate of apoptosis induced by HG&FFA (Fig. 5A). ATP content detection showed that HG&FFA stimulation reduced the intracellular ATP content by 50%, while the intracellular ATP content of the 100 μM CDP-etn group increased by 30% compared with the HG&FFA group (Fig. 5B). The JC-1 results showed that the mitochondrial membrane potential decreased significantly after HG&FFA treatment, and that of the CDP-etn group significantly increased after 100 μM CDP-etn treatment compared with the model group (Fig. 5C). Similarly, the level of ROS was significantly increased under HG&FFA stimulation, and 100 μM CDP-etn reduced the level of cellular ROS caused by HG&FFA (Fig. 5D). However, adding 100 μM LPE did not ameliorate the changes in apoptosis, ROS, ATP content, or membrane potential caused by HG&FFA. In addition, ROS levels of L02 cells under insulin resistance model were also increased (supplemental Fig. S2D). The mitochondrial oxidative capacity of L02 cells was determined using the Seahorse XF Cell Mito Stress Test. Compared to the control cells, the oxygen consumption rate of the mitochondria was significantly decreased by stimulation with HG&FFA, including basal respiration, maximal respiration, spare capacity, and ATP production (Fig. 5E). Supplementation with CDP-etn significantly ameliorated the mitochondrial function, while supplementation with LPE aggravated mitochondrial damage. These results indicate that the decrease in PE caused by the decrease in PCYT2 may be a potential cause of liver mitochondrial dysfunction in T2DM.

To determine whether supplementation with CDP-etn could also improve abnormal glucose and lipid metabolism in the hepatocytes in T2DM, we detected glucose intake and relative levels of triglyceride (TG) based on the lipidomic analysis. Our results indicated that CDP-etn-treated group, but not LPE-treated group, exhibited an increased glucose intake and decreased TG levels compared with the HG&FFA group (supplemental Fig. S4A, B).

### Overexpression of PCYT2 can ameliorate the mitochondrial damage caused by HG&FFA in hepatocytes

**Fig. 6:**
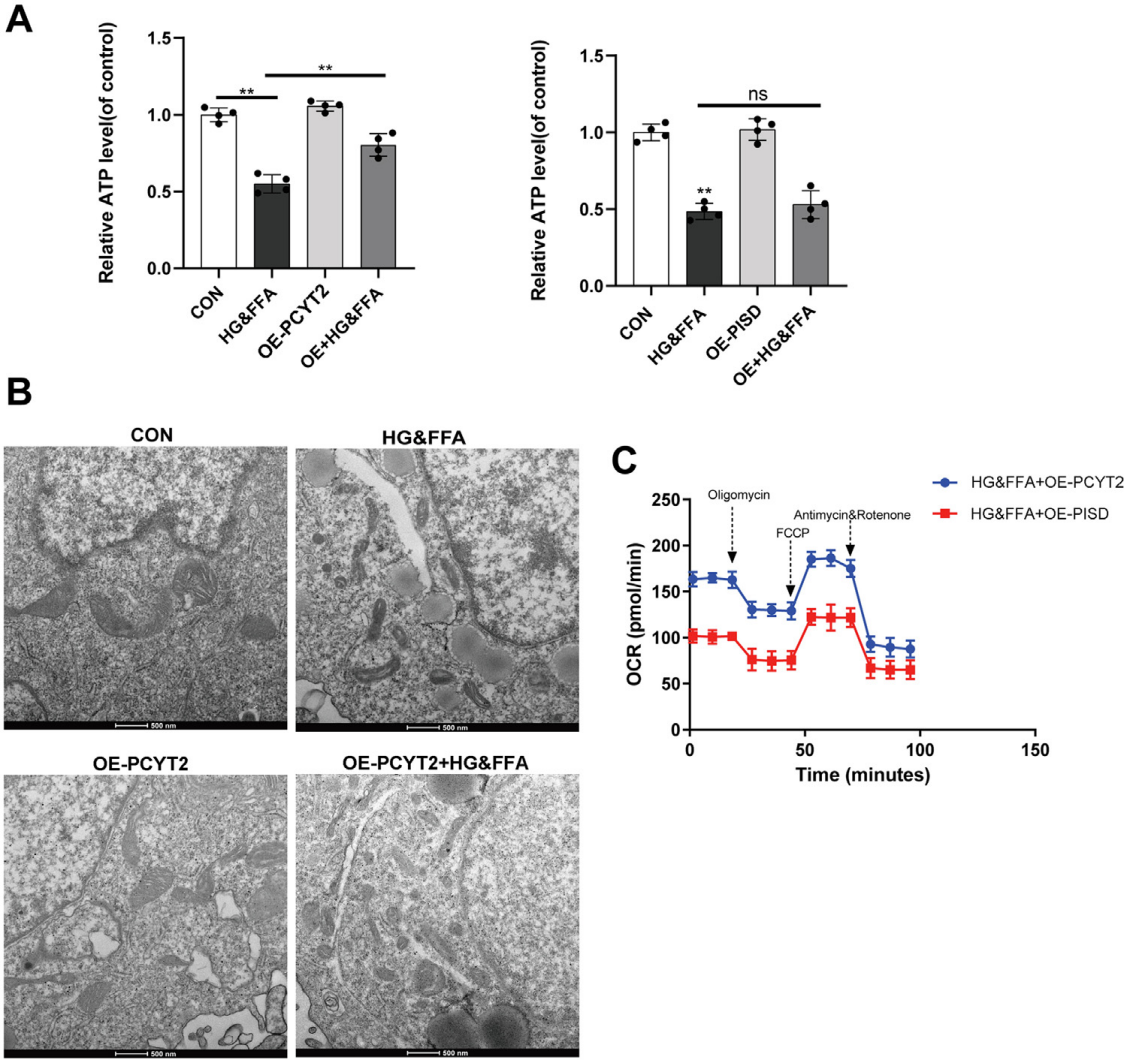
Overexpression of PCYT2 alleviated mitochondrial damage of L02 cells caused by HG&FFA. A: Changes of ATP content in L02 cells overexpressing PCYT2 or PISD (n = 3). B: Transmission electron microscopy was used to observe the ultrastructural changes of mitochondria in L02 cells. C: The mitochondrial oxygen consumption rate of L02 cells was detected using Seahorse (n = 3). All experiments were repeated three times. ∗*P* < 0.05, ∗∗*P* < 0.01. HG&FFA, high glucose and free fatty acids; PCYT2, phosphoethanolamine cytidylytransferase; PISD, phosphatidylserine decarboxylase.

To further investigate the role of PCYT2 in HG&FFA-induced apoptosis and mitochondrial damage, we tested the ATP content of L02 cells overexpressing PISD or PCYT2. The results showed that overexpression of PCYT2, but not PISD, effectively improved the reduction in ATP content caused by HG&FFA (Fig. 6A). The ultrastructure of L02 cells was observed by EM. The mitochondrial structure of cells in the HG&FFA group showed some abnormalities, including vacuoles, irregular mitochondrial membranes, and cristae breaks. OE-PCYT2 reversed the damage to the mitochondrial structure caused by HG&FFA to a certain extent (Fig. 6B). The mitochondrial oxidative capacity of L02 cells determined using the Seahorse system differed significantly between OE-PCYT2 and OE-PISD under stimulation with HG&FFA. The mitochondrial oxygen consumption rate of OE-PCYT2 cells was significantly increased compared to that of OE-PISD cells (Fig. 6C). The results showed that overexpression of PCYT2, but not PISD, could increase glucose uptake and decrease TG levels when compared with HG&FFA stimulation (supplemental Fig. S4A, B).

### PCYT2 plays an important role in apoptosis caused by HG&FFA via BAX/Bcl2/caspase3 signaling

**Fig. 7:**
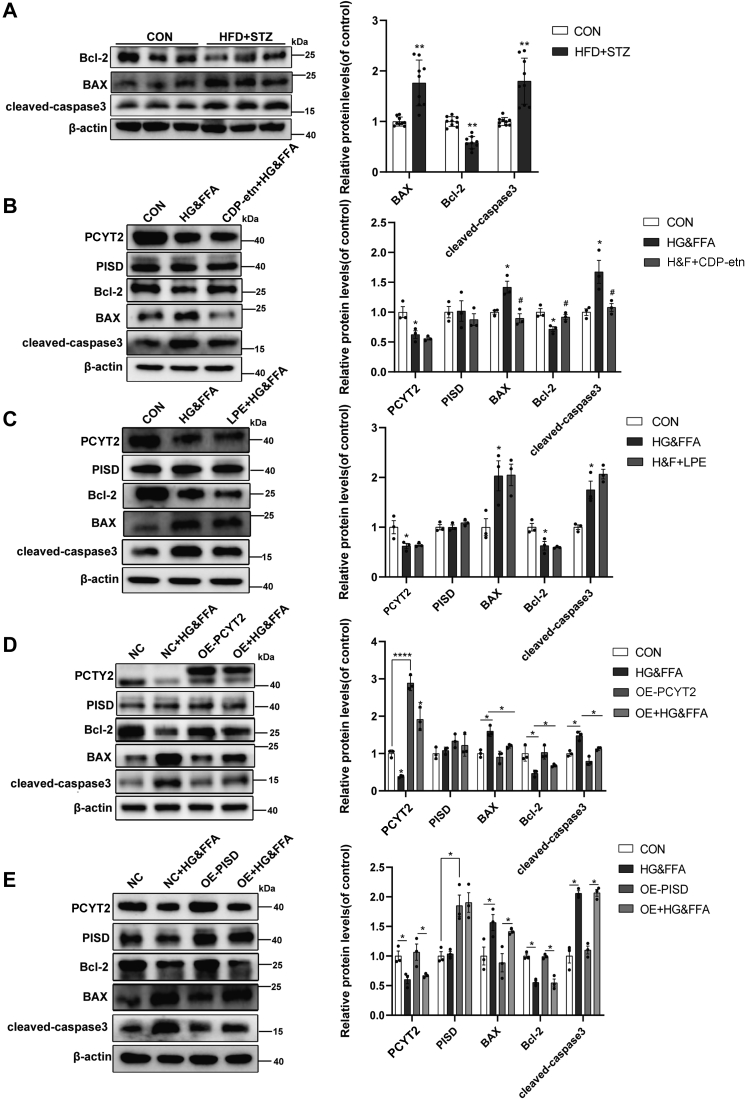
PCYT2 plays an important role in apoptosis caused by HG&FFA via BAX/Bcl2/caspase3 signaling. A: Changes of protein levels of BAX, Bcl-2 and cleaved-caspase3 in T2DM group compared with CON group in mice (n = 3). B and C, Changes of protein levels of BAX, Bcl-2, and cleaved-caspase3 in L02 cells treated with CDP-etn (B) or LPE (C) under stimulation with HG&FFA (n = 3). D and E, Protein levels of BAX, Bcl-2, and cleaved-caspase3 in L02 cells overexpressing PCYT2 (D) or PISD (E) under stimulation with HG&FFA (n = 3). All experiments were repeated three times. ∗*P* < 0.05, ∗∗*P* < 0.01. CDP-etn, CDP-ethanolamine; HG&FFA, high glucose and free fatty acids; PCYT2, phosphoethanolamine cytidylytransferase; PISD, phosphatidylserine decarboxylase; T2DM, type 2 diabetes mellitus.

To further explore the mechanism of apoptosis induced by HG&FFA in L02 cells, or by the decrease in PCYT2 in hepatocytes, we assessed the protein levels of components of apoptosis signaling, BAX, Bcl-2, and cleaved-caspase3. The levels of BAX and cleaved-caspase3 were significantly high while Bcl-2 were significantly low in the liver of HFD + STZ mice and in L02 cells after stimulation with HG&FFA. CDP-etn (100 μM) could protect cells from HG&FFA-induced apoptosis by reducing BAX and cleaved-caspase3 and increasing Bcl-2. However, such protection did not occur under 100 μM LPE treatment, which shows that the product of PCYT2 catalysis plays an important role in protecting liver cells from the effects of HG&FFA (Fig. 7A–C). The protein levels of PISD and PCYT2 in the OE-PISD and OE-PCYT2 groups increased three times and four times, respectively, compared to the controls. Under HG&FFA stimulation, compared with the HG&FFA group, the protein levels of BAX and cleaved-caspase3 in the OE-PCYT2 group decreased and the Bcl-2 protein level increased. However, such a reversal was not observed in the OE-PISD group (Fig. 7D–E). These results taken together indicate that mitochondrial damage and apoptosis of hepatocytes under HG&FFA may be caused by a decrease in PCYT2.

## DISCUSSION

Metabolomics and lipidomics studies on T2DM mice and patient serum have revealed a variety of metabolites that are present at altered levels. Among components involved in lipid metabolism, fatty acids such as myristic acid, PA, stearic acid, linoleic acid, oleic acid, and arachidonic acid are significantly increased in patients with diabetes and are thought to be independent predictors of diabetes progression as well as impair the effects of insulin. In contrast, glycerol phospholipids such as phosphatidylcholine (PC) and PE and lipid derivatives such as sheaths dihydroammonia and aminol are reduced in diabetic patients, as some other metabolites are involved in carbohydrate metabolism, amino acid metabolism, and the tricarboxylic acid cycle. These changes are considered important clinical markers (25, 26, 27). Although previous studies reported changes in these metabolites, they did not explore the molecular mechanisms underlying these changes.

**Fig. 8:**
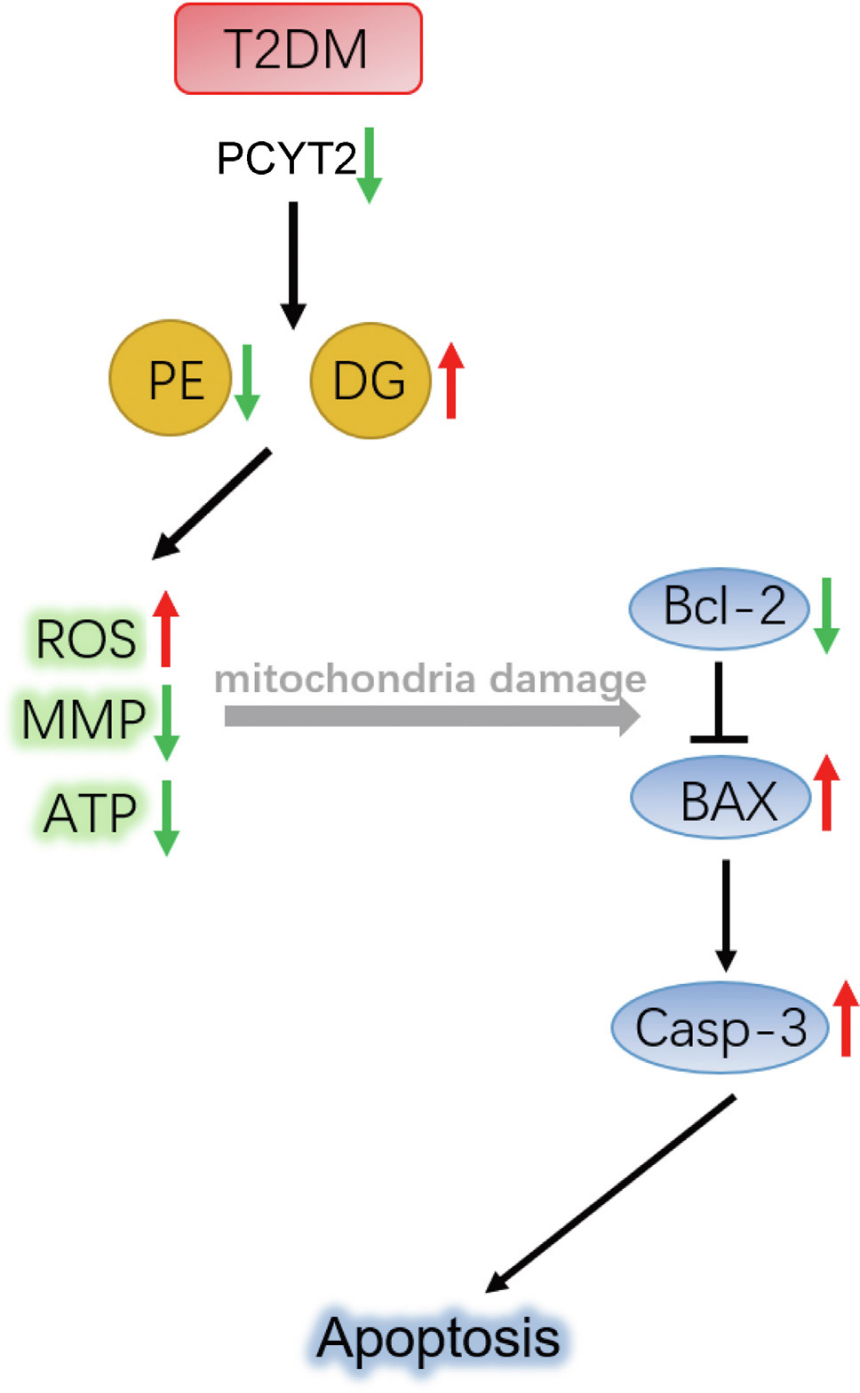
In type 2 diabetes, decreased expression of PCYT2 in liver leads to decreased PE content and increased DG content, resulting in decreased mitochondrial membrane potential, increased production of reactive oxygen species, reduced ATP content and other mitochondrial damage, thereby activating the apoptosis pathway of BAX/Bcl-2/cleaved-caspase3, thus cause apoptosis of liver cells, resulting in liver damage. DG, diglyceride; PCYT2, phosphoethanolamine cytidylytransferase; PE, phosphatidylethanolamine.

Our study has demonstrated that the PE content in the livers of T2DM mice is significantly reduced and the mechanism of this reduction may be a decrease in Pcyt2. Our study has further shown that the reduction of PE also causes mitochondrial dysfunction, including decreased mitochondrial membrane potential, decreased ATP content, increased ROS, and increased apoptosis, which may be mediated by activation of the BAX/Bcl-2/caspase3 pathway (Fig. 8).

PE plays an important regulatory role in neurodegeneration and the occurrence and development of some neurodegenerative diseases; the lack of PE affects the progression of Alzheimer’s disease and Parkinson’s disease (13). PE is thought to be a target of ophiobolin A, a powerful natural anticancer agent. Therefore, PE is thought to play an important role in killing tumor cells. Moreover, LACTB, a tumor suppressor gene, is downregulated in many tumor cell lines and has been found to downregulate PISD. These results open up a new field of PE research for the future (28). PCYT2 is a rate-limiting enzyme in the CDP-etn pathway; knocking it out can result in a variety of effects. A systemic knockout of *Pcyt2* in mice is embryonic lethal, and knocking out *Pcyt2* in the liver causes severe TG deposition. Knocking out *Pcyt2* in muscle tissue will not only cause a significant increase in DG and TG content but also an increase in mitochondrial content, both of which will also cause a significant decrease in PE (19, 29, 30). These findings indicate that PCYT2 plays an important role in regulating lipid and energy homeostasis. However, the role of PCYT2-mediated energy metabolism in liver injury induced by T2DM is not well understood.

T2DM patients often exhibit impaired energy metabolism, which is characterized by mitochondrial dysfunction, high production of ROS, and low levels of ATP (31, 32). The mitochondrial apoptotic pathway is activated in hepatocytes of patients with T2DM (33). Our study suggests that the reduction of PE from the CDP-etn pathway in T2DM may also induce apoptosis through the BAX/Bcl-2/caspase3 pathway. After supplementing with CDP-etn, a direct product of PCYT2, or by overexpression of PCYT2, some damage caused by HG&FFA was reversed, providing evidence in support of our hypothesis. Combined with the lipidomics results, these results further prove that PE is an indispensable participant in cell survival and that PCYT2 plays an important role in regulating cell survival. This provides a new area for future research on lipids in T2DM.

However, our study had certain limitations. DG is believed to play a role in promoting insulin resistance (34). Although we found a significant increase in the content of DG, we have not yet investigated whether the increase in DG can further aggravate insulin resistance and increase liver damage in the diabetic model. Moreover, the specific mechanism underlying the decrease in PCYT2 expression in T2DM is still unknown. Studies have shown that the upstream transcription factors of PCYT2 may include early growth response factor 1 (EGR1), E74 like ETS transcription factor 3 (ELF3), and nuclear transcription factor Y subunit alpha (NFYA) (35, 36, 37). EGR1 promotes the progression of nonalcoholic fatty liver disease in patients with insulin resistance (38). NFYA is an important transcriptional regulator of lipid and glucose metabolism and adipokine biosynthesis related to the occurrence of T2DM (39). However, our qRT-PCR results showed that EGR1 in vitro and in vivo and ELF3 and NFYA in T2DM in vitro did not change significantly (data not shown). To further comprehend the complexity of diabetes, the changes in PCYT2 transcription factors in the liver of T2DM patients need further study.

Interestingly, PE is the most abundant among all phospholipids in mitochondria, and PE in mitochondria is synthesized by the PISD pathway (40). In our study, no significant changes were observed in the PISD; this might be because the activity of PISD may be prevalent in muscles. The main synthetic pathway of PE in the liver is the CDP-etn pathway, which can also be seen by the difference in the composition of PE between the two in different organs (41, 42). In addition, because there is a unique PC synthesis pathway in the mammalian liver, PE is catalyzed by PE N-methyl transferase to form PC, which provides 30% of the PC content in the liver. The PC/PE ratio is a determinant of the oxidative capacity and energy production of the liver mitochondria. This may explain why the reduction of PE outside the mitochondria also affects the normal function of mitochondria. Similarly, PE in the phospholipid remodeling pathway can also be transferred to mitochondria through the generation of PS by PSS2, which may also affect the generation of mitochondrial PE pool as the raw material for PE synthesis in PISD, thus affecting mitochondrial oxidative phosphorylation ability (14, 43). It is worth noting that the decrease in PCYT2 often leads to the accumulation of the upstream product phosphoethanolamine, a metabolite that damages cellular respiration (19). However, whether the reduction of PCYT2 in T2DM leads to the accumulation of phosphoethanolamine and affects mitochondrial respiration requires verification, but we still believe that the reduction of PE also leads to mitochondrial dysfunction. In addition, other phospholipids may also play a role in T2DM and regulate insulin sensitivity. For example, PC and phosphoinositol are associated with insulin resistance. However, the specific functions of these phospholipids in T2DM and the regulation of phospholipid remodeling in the disease need to be further explored (44, 45, 46).

In summary, we have demonstrated the reduction of PE in the liver in T2DM and its possible mechanism, which emphasizes the importance of PE produced by the PCYT2 pathway and opens new avenues for the treatment of liver injury in patients with T2DM in the future.

## Data availability

All data described are included in the manuscript and the supplementary data.

## Supplemental data

This article contains supplemental data.

## Conflict of iInterest

The authors declare that they have no conflicts of interest with the contents of this article.
