# Supplementary material for: Phosphoethanolamine cytidylyltransferase ameliorates mitochondrial function and apoptosis in hepatocytes in T2DM in vitro
Source: J Lipid Res. 2023 Jan 28;64(3):100337. doi: 10.1016/j.jlr.2023.100337 (PMC10033998; doi:10.1016/j.jlr.2023.100337)
Supplement: Supplemental data [file mmc1.pdf]

Table S1. List of all primer sequences used

Name	Sequence (forward)	Sequence (reverse)
**Mouse**		
Pcyt2	CTATGACATGGTGCATTATGGC	CTGTACCATCTTGTACCTCTCC
Pisd	TCAGTCAGAGAAGCAGCCAGGAC	CAGGAGGTAGTGGAGGATGGTCAG
Egr1	CCCAGGACTTAAAGGCTCTTAA	TGGTCACTACGACTGAAGTTAC
Actb	CCTGGCACCCAGCACAAT	GGGCCGGACTCGTCATAC
**Human**		
PCYT2	CACGGCAAGACAGAAATTATCC	TTGGCTTCCTTCTTCTGGTTTC
PISD	CTTTGTACAAGTCAGTGCCAAC	CCAGATGTACAGGCTGTAGAC
EGR1	CAGCAGCACCTTCAACCCTCAG	CCACCAGCACCTTCTCGTTGTTC
ELF3	ATGGTTTTCGTGACTGCAAGAA	CAGTACTCTTTGCTCAGCTTTC
NF-YA	GATTGTTCAAACAGGAGCCAAT	AGAGGGATTCTTTGGATAGCAG
ACTB	GTGCTATGTTGCTCTAGACTTCG	ATGCCACAGGATTCCATACC

A

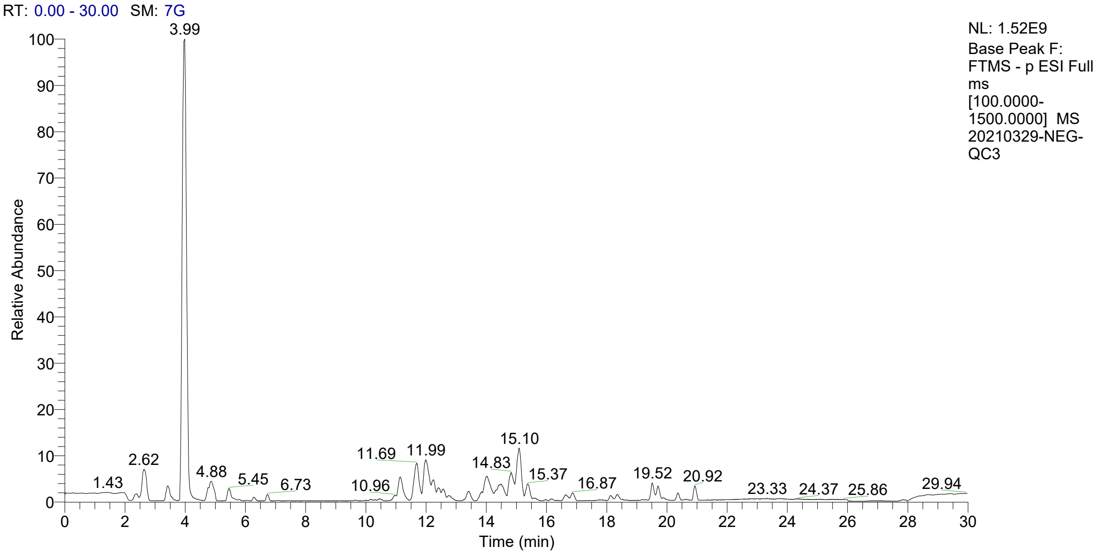

B

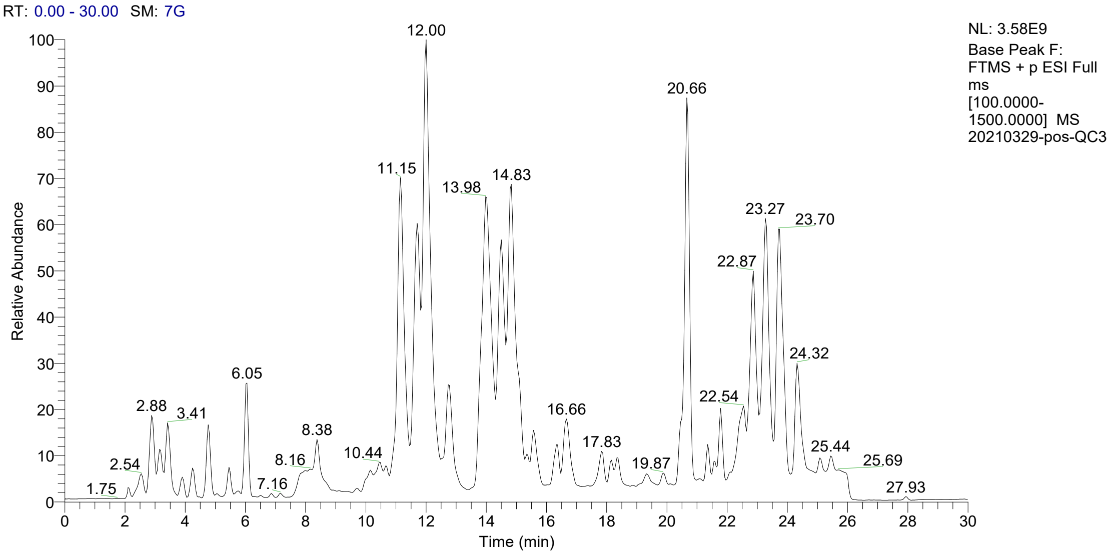

Figure S1. Related to Figure 1. Typical base peak chromatogram of the QC sample in ESI^- (A) and ESI^+ (B) modes based on the mice liver lipidomic profile.

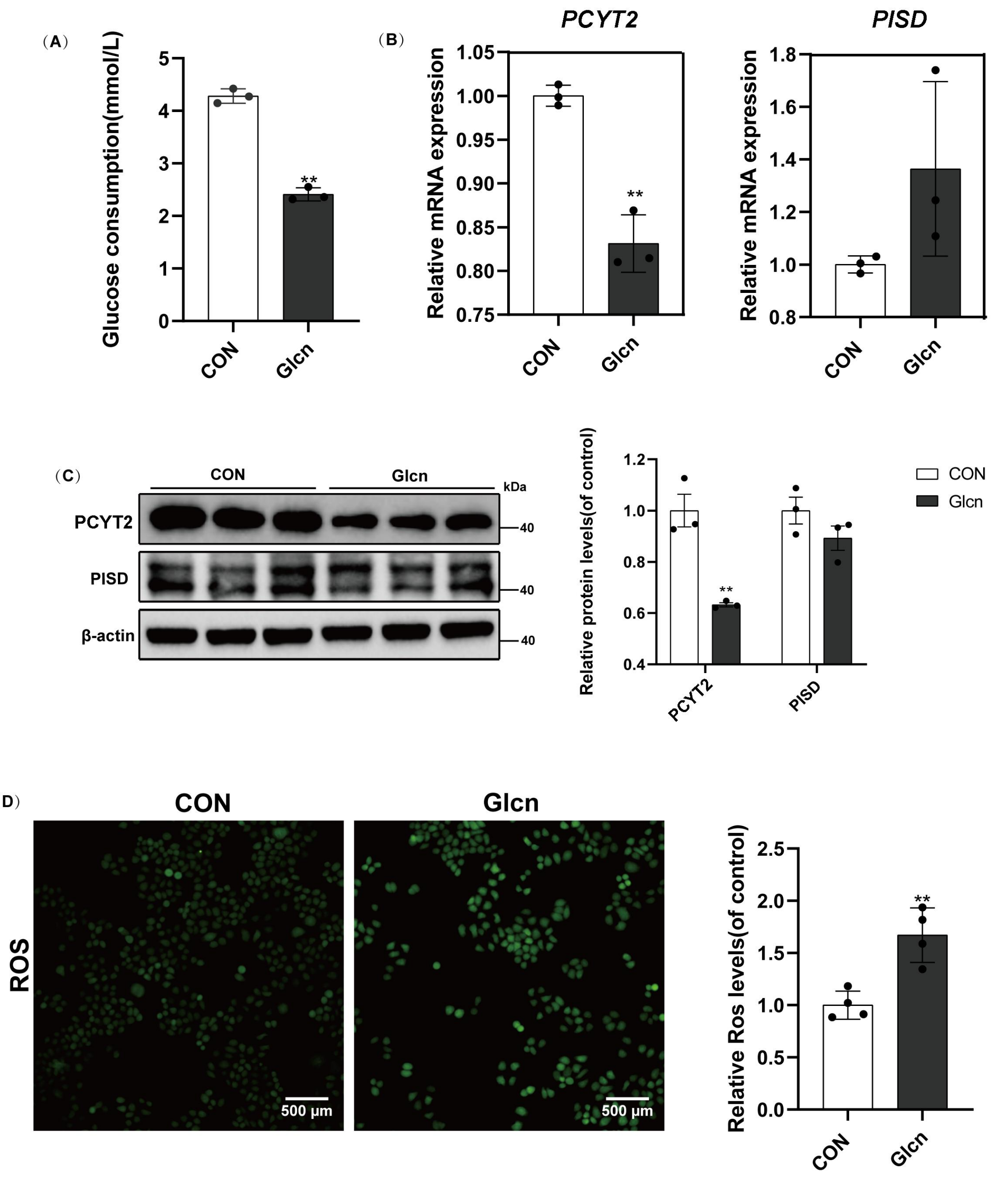

Figure S2. Related to Figure 3 and Figure 5. (A) Glucose consupmption in L02 cells treated with glucosamine or not. (B) *PISD* and *PCYT2* mRNA levels in L02 cells treated with glucosamine or not. (C) PISD and PCYT2 protein expression levels in L02 cells treated with glucosamine or not. (D) ROS levels in L02 cells treated with glucosamine or not.

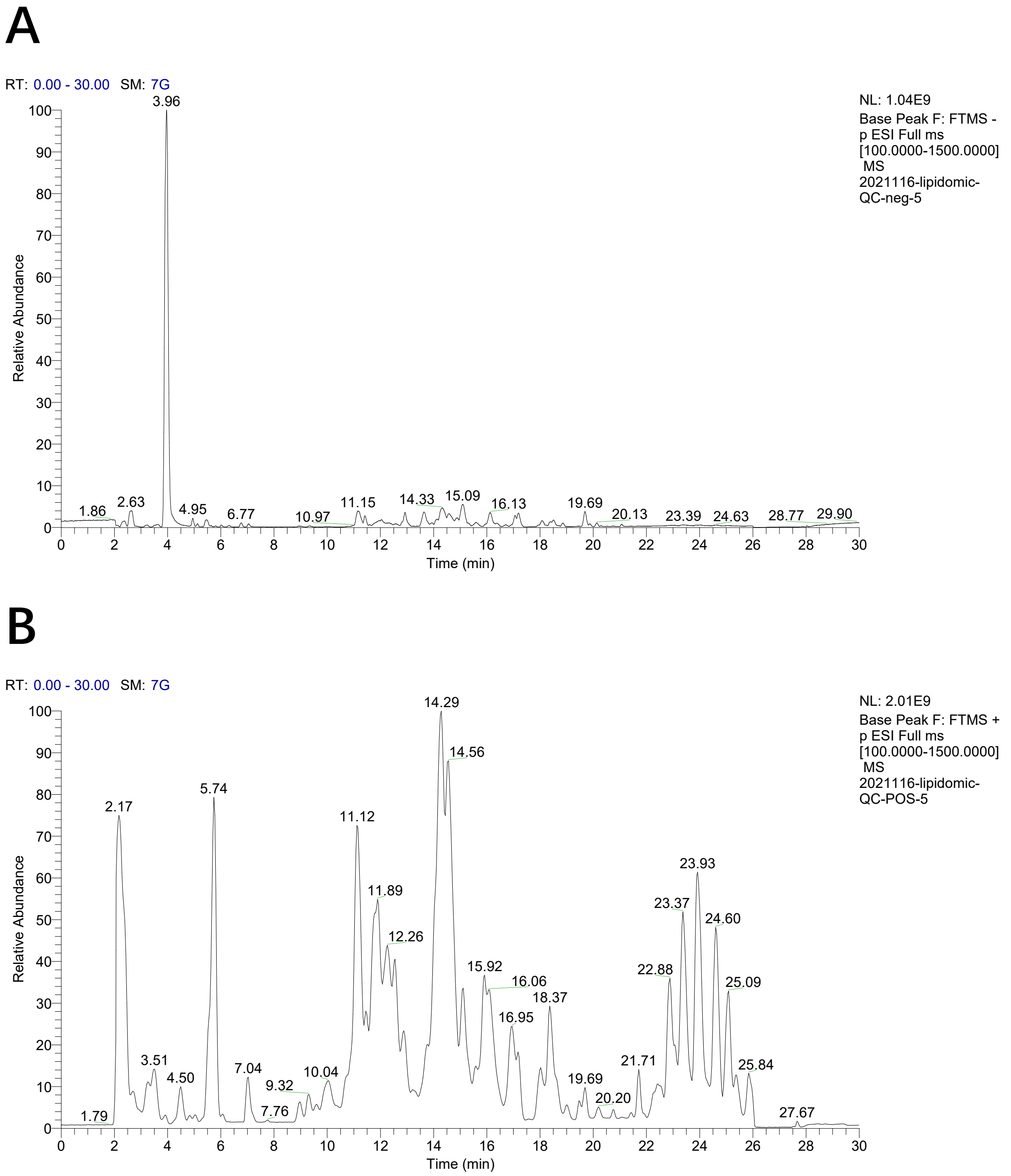

Figure S3. Related to Figure 4. Typical base peak chromatogram of the QC sample in ESI^- (A) and ESI^+ (B) modes based on the L02 cells lipidomic profile.

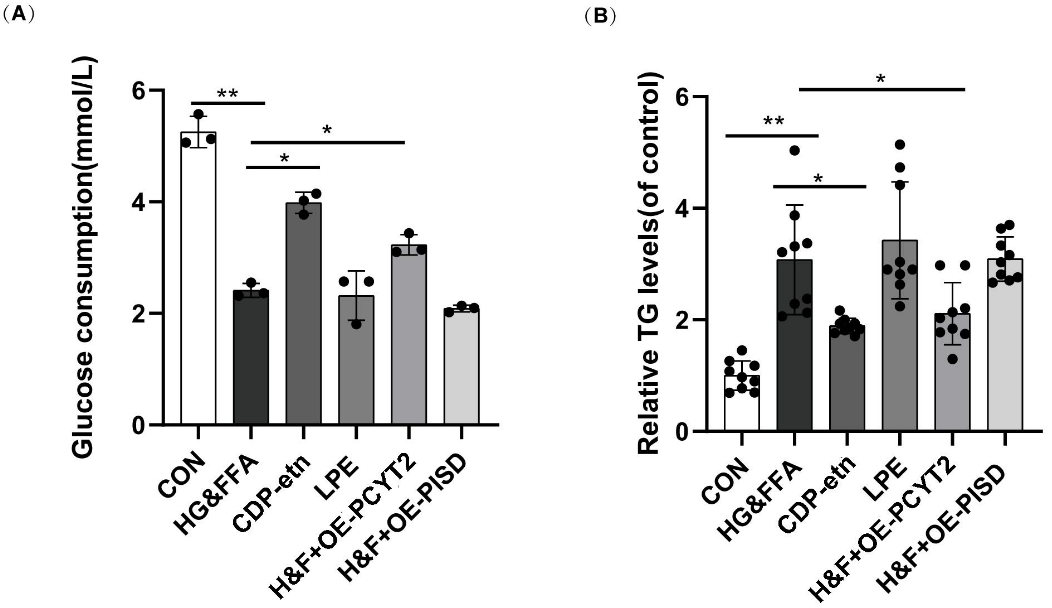

Figure S4. Related to Figure 5 and Figure 6. The glucose uptake and TG relative levels based on lipidomic analysis in different groups of cells (CON, control group; HG&FFA, high glucose and free fatty acids; CDP-etn, supplement of CDP-etn in L02 cells stimulated with HG&FFA; LPE, supplement of LPE in L02 cells stimulated with HG&FFA; H&F+OE-PCYT2, overexpression of PCYT2 in L02 cells stimulated with HG&FFA; H&F+OE-PISD, overexpression of PISD in L02 cells stimulated with HG&FFA. *P <0.05, **P < 0.01
